# Microglia in the Mouse Retina Alter the Structure and Function of Retinal Pigmented Epithelial Cells: A Potential Cellular Interaction Relevant to AMD

**DOI:** 10.1371/journal.pone.0007945

**Published:** 2009-11-20

**Authors:** Wenxin Ma, Lian Zhao, Aurora M. Fontainhas, Robert N. Fariss, Wai T. Wong

**Affiliations:** 1 Unit on Neuron-Glia Interactions in Retinal Disease, National Eye Institute, National Institutes of Health, Bethesda, Maryland, United States of America; 2 Biological Imaging Core, Office of the Scientific Director, National Eye Institute, National Institutes of Health, Bethesda, Maryland, United States of America; University of Oldenburg, Germany

## Abstract

**Background:**

Age-related macular degeneration (AMD) is a leading cause of legal blindness in the elderly in the industrialized word. While the immune system in the retina is likely to be important in AMD pathogenesis, the cell biology underlying the disease is incompletely understood. Clinical and basic science studies have implicated alterations in the retinal pigment epithelium (RPE) layer as a locus of early change. Also, retinal microglia, the resident immune cells of the retina, have been observed to translocate from their normal position in the inner retina to accumulate in the subretinal space close to the RPE layer in AMD eyes and in animal models of AMD.

**Methodology/Principal Findings:**

In this study, we examined the effects of retinal microglia on RPE cells using 1) an *in vitro* model where activated retinal microglia are co-cultured with primary RPE cells, and 2) an *in vivo* mouse model where retinal microglia are transplanted into the subretinal space. We found that retinal microglia induced in RPE cells 1) changes in RPE structure and distribution, 2) increased expression and secretion of pro-inflammatory, chemotactic, and pro-angiogenic molecules, and 3) increased extent of *in vivo* choroidal neovascularization in the subretinal space.

**Conclusions/Significance:**

These findings share similarities with important pathological features found in AMD and suggest the relevance of microglia-RPE interactions in AMD pathogenesis. We speculate that the migration of retinal microglia into the subretinal space in early stages of the disease induces significant changes in RPE cells that perpetuate further microglial accumulation, increase inflammation in the outer retina, and fosters an environment conducive for the formation of neovascular changes responsible for much of vision loss in advanced AMD.

## Introduction

Age-related macular degeneration (AMD) is the leading cause of legal blindness and visual disability for individuals aged 60 and over in the Western hemisphere[1]–[4]. While the pathogenesis of AMD is still unknown, multiple early disease alterations have been noted to involve the retinal pigment epithelium (RPE) monolayer, a highly specialized layer of epithelial cells essential for supporting photoreceptors and maintaining the outer blood retina barrier [5]. In early and intermediate stages of AMD, lipoproteinaceous deposits called drusen collect beneath the RPE layer [6]. In addition, RPE cells develop changes in structure and distribution that are evident as patchy hyper- and hypopigmentary changes in the fundus (so-called “pigment mottling”)on clinical examination [7], and also as RPE depigmentation, hypertrophy, hyperplasia, and atrophy, as seen on histopathological examination [8]. As AMD progresses, choroidal neovascularization (CNV) (or “wet” AMD) can arise when new blood vessels from the choroid invade the basement membrane of the RPE layer, the Bruch's membrane, and extend into the outer retina, with ensuing photoreceptor cell atrophy and vision loss [9]. While early structural changes in the RPE layer are often considered a harbinger of AMD disease progression [10], the initiating cellular and molecular factors inducing pathogenetic alterations in the RPE are incompletely understood.

The role of inflammation has been prominently implicated in the pathogenesis of AMD [11], [12]. The subretinal space, the interface between the apical surface of the RPE and the outer segments of photoreceptors, is a locus of particular interest in the relationship between inflammation and AMD. Under normal conditions, the subretinal space is a zone of special immune privilege[13], maintained by the activity of RPE cells which secrete immunosuppressive factors into this space[14], and also by the notable absence of retinal microglia, which perform dynamic immune surveillance in the inner retina, but are largely excluded from the outer retina [15]. However, under conditions of advanced age and photoreceptor injury, retinal microglia translocate into the outer retina [16], accumulate in the subretinal space [17], and acquire morphological features of activation [18]. Significantly, activated microglia have also been found in the subretinal space of patients with AMD [19], and are juxtaposed in close proximity with RPE cells overlying drusen (Wong and Fariss, unpublished data). Additionally, in a number of mouse models for AMD, involving the absence of chemokine ligands/receptors, CCL2 and/or CX3CR1 [20], [21], microglia accumulation in the subretinal space was accelerated and more pronounced. This accumulation of subretinal microglia was in turn associated with multiple features reminiscent of AMD histopathology including the accumulation of drusen-like deposits, local RPE structural changes, and CNV formation [20], [21].

The anatomical separation between retinal microglia and RPE cells under normal conditions and their juxtaposition into direct and intimate contact in the subretinal space under pathological situations suggest that cellular interactions between these two retinal cell types may be of particular pathogenetic significance. Microglia, serving as resident immune cells of the retina, can have multiple functional states and carry out diverse functions [22]. Capable of rapid dynamism and motility, they also synthesize and release multiple cytokines, chemokines, neurotrophic factors, and neurotransmitters that allow them to interact with multiple CNS cell types and exert cytotoxic or cytoprotective effects depending on the tissue context [23]. While there has been evidence that retinal microglia may interact with signals from retinal neurons [24], photoreceptors [25], and retinal vessels [26], [27], the direct influences of retinal microglia on RPE structure, physiology, and gene expression have not been previously addressed. An understanding of the nature of this cell-cell interaction has the potential to shed useful light on the cellular basis of the inflammatory etiology of AMD.

In this study, we examined the nature of microglia-induced effects on RPE cells using an *in vitro* co-culture model where cultured activated mouse retinal microglia were co-cultured with primary RPE cells from animals of the same genetic background. We examined the consequences of microglial co-culture on RPE structure, physiology, and the expression and secretion of molecules important in influencing the inflammatory and angiogenic environment in the subretinal space. In addition, we employed an *in vivo* model of microglia-RPE interactions by transplanting cultured microglia into the subretinal space of experimental mice. Our results show that signals from activated retinal microglia prominently alter the structure, properties, and gene expression of RPE cells both *in vitro* and *in vivo*. These alterations constitute a disorganization of the RPE monolayer and create a pro-inflammatory environment in the subretinal space conducive for the further recruitment of retinal microglia and for the formation of CNV. The similarities between these alterations and the important clinical features found in AMD strongly suggest that microglia-RPE interactions play an important and determining role in the pathogenesis of AMD. Microglia-RPE interactions may represent a potential locus of therapeutic intervention in the treatment and prevention of vision loss in AMD.

## Methods

### Culture of Mouse Retinal Microglia Cells

All animals were handled in strict accordance with good animal practice as defined by the relevant national and/or local animal welfare bodies, and all animal work was approved by the NEI Institutional Animal Care and Use Committee. Experimental animals were housed and bred in National Institutes of Health animal facilities. Microglia were isolated from retinas of young C57BL/6 mice (postnatal day (P)10–30) using a method modified from Roque and Caldwell [28]. Wild-type C57BL/6 mice, as well as transgenic mice in which one copy of the CX3CR1 gene has been replaced by the gene coding for green fluorescent protein (CX3CR1^+/GFP^, strain B6.129 ^Cx3cr1tm1/litt/^J, obtained from Jackson Labs, Bar Harbor, ME) [29], were used.

Briefly, experimental animals were euthanized and their eyes enucleated. The globes were dissected free of periocular connective tissue, rinsed with HBSS buffer, transferred into 2% dispase (Roche, Nutley, NJ) in HBSS, and placed in an incubator at 37°C for 45–60 minutes. Dispase activity was terminated by washing the globes three times in DMEM (high glucose and L-glutamine) plus 10% FBS (Gibco/Invitrogen, Carlsbad, CA). The anterior segment and vitreous were excised, and the retina dissected free from the underlying RPE layer. RPE-sclerochoroidal eyecups were set aside for the primary culture of RPE cells. The retinas were transferred into culture medium consisting of DMEM with high glucose, 10% FBS, 1x MEM non-essential amino acids solution (Sigma, St Louis, MO), and subsequently triturated several times with a transfer pipette. The dissociated cells were transferred into 75 cm^2^ flasks and left to grow in a 37°C incubator. After the mixed culture had grown confluent, microglia were found distributed on the top of the cell layer and could be detached by shaking the flask by hand. The detached cells, composed of 95% microglia, were then cultured in 150 mm dishes at low density in the same culture medium. Each microglia cell, separated from its neighbors at low culture densities, divided over the next 2–3 weeks to form a cluster of adherent cells. Individual cell clusters comprised solely of microglia (identifiable by their branching morphology and their GFP-positivity when isolated from CX3CR1^+/GFP^ retinas) were picked off the culture dish with a sterile pipette and cultured in a new 25 mm^2^ flask. The purity of microglia in this resulting culture exceeded >98% and these cultures were used in subsequent co-culture and *in vivo* transplantation experiments.

### Primary Culture of Mouse RPE Cells

Isolated RPE-sclerochoroidal eyecups from young adult C57Bl6 mice were obtained as described above. The loosely adherent RPE cell layer was gently peeled off each eyecup using fine forceps, transferred to a 15 ml tube containing culture medium comprised of Medium199, 15% FBS (Gibco, Carlsbad, CA) and 1x MEM with non-essential amino acids (Sigma, St. Louis), and then triturated with a transfer pipette to form a dissociated cell suspension. This suspension was plated on a serum pretreated 6-well plate. RPE cells from a total of 10–12 eyes were used per 6-well plate. The medium was changed after 7 days, and every 2–3 days thereafter. The RPE cells grew to form a confluent cell layer by 2–3 weeks and were used in co-culture experiments at that point.

### Co-culture of Retinal Microglia Cells with Primary RPE Cells

Cultured retinal microglia were collected and cultured onto Transwell permeable support membrane inserts (Corning, Corning, NY) and grown to 60–70% confluence. The microglial cultures were first incubated in DMEM with 5% heat-inactivated serum for 24 hrs, then activated by the addition of lipopolysaccharide (LPS) (1 µg/ml, Sigma) for 6 hrs, and subsequently washed well 3 times in HBSS to remove all traces of LPS. Separately, RPE cultures were grown to confluence in a 6-well plate (density of 1-5×10^6^ cells/well), washed 3 times with HBSS, and then placed in 0.5 ml of DMEM with 5% heat-inactivated serum. The previously prepared Transwell inserts containing either untreated or LPS-treated retinal microglia were then placed into wells with RPE cells with 1 ml of DMEM with 5% heat-inactivated serum. Control RPE cultures were incubated with empty inserts without cultured microglia. RPE-microglia co-cultures and controls were kept in a 37°C incubator in 5% CO_2_ for 24 hours. At the end of this period, the inserts were removed, and the supernatant overlying the RPE cells collected. The RPE cells in the well were washed twice with PBS and then homogenized for mRNA or protein analysis. At least 3 co-culture repeats were performed for all analyses.

### Immunohistochemistry

Cultured cells and retinal tissue for immunohistochemical analyses were fixed (30 minutes for cultured cells and 4 hours for retinal tissue) in 4% paraformaldehyde in PBS, washed with phosphate buffered saline containing 0.25% Tween 20 (PBST), and then permeabilized with Triton-X (0.25% for cultured cells, 1% for tissues)(Sigma, St. Louis, MO) for 30 minutes. Cells or tissues were placed in a blocking solution (USB, Rodeo Blocker, Cleveland, OH) for 30 minutes, and incubated overnight with primary antibodies to the following antigens: CD11b, 1∶200; F4/80, 1∶200; CD68, 1∶200 (all from AbD Serotec, Raleigh, NC); ionized calcium binding adaptor molecule-1 (Iba1), 1∶500 (Wako, Richmond, VA); GFAP, 1∶200; Alexa Fluor 633-conjugated Phalloidin, 1∶100; zonula-occludins-1 (ZO-1), 1∶200 (all from Invitrogen, Carlsbad, CA); NeuN, 1∶200 (Millipore, Billerica, MA); vimentin, 1∶200 (MP Biomedicals, Solon, OH); CD31 [P2B1], 1∶100 (Development Studies Hybridoma Bank, Iowa City, IO). After washing, secondary antibodies, which were conjugated to either Alexa-488 or Alexa-568 (Invitrogen, Carlsbad, CA), were added at a 1∶200 dilution and incubated for 1–2 hrs.

### Semi-Quantitative RT-PCR

Total RNA was extracted from RPE cells with RNeasy Mini kit (Qiagen, Valencia, CA). First-strand cDNA was synthesized with a cDNA synthesis kit (Ambion, Austin, TX) according the manufacturer's protocol using oligo-dT as the primer. PCR amplifications were conducted in a total volume of 15 µl, comprising of 1 µl of the cDNA, 2 µl of primer mixture, and 12 µl of Plus Master Mix (Qiagen, HotStarTaq Plus). After an initial denaturation at 95°C for 5minutes, PCR amplification was conducted for 20–35 cycles, and GAPDH was used as an internal control. A list of primers used is provided in Table 1. At least 3 replicates of each experiment were performed.

**Table 1:** DNA primers used for RT-PCR.

Oligos	Forward (5′- 3′)	Reverse (5′- 3′)	Tm(°C)	Length(bps)
Claudin1	gcagctgctgggtttcatc	gtctttcccactagaaggtg	56	610
CRALBP	gagtggttatgctcttcaacatc	cataaggctgtgttctcaacttc	56	467
CX3CL1	ttcacgttcggtctggtggg	ggttcctagtggagctaggg	60	970
GAPDH	cctctggaaagctgtggcg	gttgctgtagccgtattcatt	56	404
GM-CSF	gtggctgcagaatttacttttc	ggactggttttttgcattcaaa	56	416
ICAM1	ctgaccctgagccagctgg	ggcctcctcctgagccttc	56	633
IFN-γ	ctggtggttgctcctcttac	ctcctgggcctctcctgtg	56	674
IL10	ggctcagcactgctatgct	gatcatcatgtatgcttctatg	56	516
IL1-β	gccaccttttgacagtgatgag	ttaggaagacacagattccatg	56	778
IL6	atgaagttcctctctgcaagag	ctaggtttgccgagtagatctc	56	636
MCP1	gcaggtccctgtcatgctt	ctagttcactgtcacactgg	56	445
MMP1a	ggtaaatagattcatgccagaac	ccatgtactagaatcacggg	56	478
MMP1b	ggttctacattcgggtaattagc	cactggaatcatcaattcttcttg	56	477
MMP2	ggaccctggagctctgatg	ccagccagtctgatttgatgc	56	737
MMP9	cctcacctggccccacaag	ctgcaggaggtcgtaggtc	56	713
Occludin	ctgactatgcggaaagagttg	ccgtctgtcataatctcccac	56	626
OTX2	ccgccaacagcagcagcag	cctggaatttccatgaggac	56	584
PEDF	ccagtgccctcagcatcctt	ccaggattctgcctatgaagag	56	642
Rantes	gaagatctctgcagctgccc	ctagctcatctccaaatagttg	56	274
RPE65	gccaatttacgtgagaattggg	cagtccatggaaggtcacag	56	558
SDF-1α	ggacgccaaggtcgtcgc	cttgtttaaagctttctccagg	56	265
TGFβ	ccactgatacgcctgagtg	gctgcacttgcaggagcg	56	605
TNFα	atgagcacagaaagcatgatc	tcacagagcaatgactccaaag	56	678
VCAM1	ggataccagctcccaaaatcc	cactttggatttctgtgcctc	56	605
VEGF	ctactgccgtccgattgagac	ggcttgtcacatctgcaagtac	56	417
ZO1	gctcatagttcaacacagcctc	ggtcaatcaggacagaaacaca	56	511

### ELISA and Western Blotting

Following co-culture with retinal microglia, the media of the RPE cell cultures were collected for analysis. Also, RPE cells at the bottom of the culture wells were then washed 3 times with PBS and homogenized in 300 µl of RIPA buffer (Sigma, St. Louis) containing a protease inhibitor cocktail (Calbiochem, Gibbstown, NJ). The cell lysate was transferred into a 1.5 ml eppendorf tube, incubated on ice for 20 minutes, and centrifuged for 20 minutes at 16,000 rpm. The clarified RPE cell lysates were then analyzed by Western blotting and ELISA. The overall protein level in both RPE lysates and RPE co-culture media were measured with BCA Protein Assay kit (Pierce, Rockford, IL). Pigment epithelium-derived factor (PEDF) and CX3CL1 protein levels were measured with ELISA kits (BioProducts MD, Middletown, MD and R&D Systems, Minneapolis, MN) according to the manufacturers' instructions. The levels of MMP1 protein were evaluated by Western blotting. The levels of all other proteins were assayed with a commercial chemiluminscence-based ELISA (Searchlight, Aushon Biosystems, Billerica, MA). At least 3 replicates of each experiment were performed.

Western blots for MMP1 were performed by first diluting the samples 1∶5 with PBS, and loading 15 µl of the solution on a NuPAGE 4–12% Bis-Tris gel (Invitrogen). Following electrophoresis, proteins in the gel were transferred onto nitrocellulose membranes (iBlot Gel Transfer Stacks, Invitrogen). After blocking overnight with a buffer containing 3%BSA and 5% blocking reagent (Rodeo Blocker, USB), the blot was incubated with the primary antibodies for MMP1 (1∶1000) (Abbiotec, San Diego, CA) or β-actin (1∶2000) (Santa Cruz Biotechnology, Santa Cruz, CA) for 1 hr. Anti-rabbit-HRP (IVD Research, Carlsbad, CA) or anti-goat HRP (Santa Cruz Biotechnology) were used as secondary antibodies (1∶3000, 1 hr). Resulting blots were developed using chemiluminescent detection (Amersham Hyperfilm ECL, GE Healthcare, Piscataway, NJ) and protein expression levels quantitated using image analysis software (ImageQuant, GE Healthcare).

### RPE Apoptosis and Proliferation Assays

RPE cells from co-culture experiments and their controls were fixed with 4% paraformaldehyde in PBS for 30 minutes and incubated in 0.25% Triton X-100 for 30 minutes. Apoptotic cells were labeled using a TUNEL assay kit according to the manufacturer's instructions (Roche, Nutley, NJ), imaged using an epifluorescence microscopy, and counted. Quantification of BrdU labeling was used to measure RPE proliferation. BrdU was added to the cultures to a concentration of 10 µM and incubated for 2.5 hours after which the cells were fixed in 4% paraformaldehyde in PBS. Proliferating cells were stained with an anti-BrdU antibody overnight (G3G4, 1∶200, Developmental Studies Hybridoma Bank, Iowa City, IA), followed by an anti-mouse secondary Alexa-568 antibody (Invitrogen), and BrdU+ cells counted in representative 10x fields.

### Angiogenesis Assays

Primary endothelial cell proliferation was measured using a BrdU labeling method. Endothelial cells from mouse aorta were grown at low density in collagen-coated chambers in experimental and control co-culture RPE supernatants for 3 days. BrdU was added to the cultures to a concentration of 10 µM and incubated for 2.5 hours, after which the cells were placed in 4% paraformaldehyde in PBS for 30 min, 0.5% triton X-100 for 30 min, 1∶1 solution of PBST and 4N HCl for 1 hr, and then a 2% blocking agent for 30 min. Labeling and quantification of proliferating cells were performed as described earlier for RPE cultures.

*In vitro* endothelial cell proliferation and migration were also assessed using the scratch wound healing assay [30]. Briefly, primary mouse aortic endothelial cells were grown to confluence on 6-well tissue culture plates whereupon a scratch wound of uniform width was made with a sterile 200-µl pipette tip through the center of the well. After wounding, the cultures were washed 3 times with serum-free DMEM medium. One ml of conditioned medium from experimental and control co-cultures were then added to each well. The cells in each well fixed and stained with CD31 and DAPI at 0 and 16 hr post-wounding, photographed with an inverted microscope, and the width of the scratch wound (mean of 6 measurements per scratch wound) measured. At least 9 scratch assays were performed per condition.

Sprouting angiogenesis in an organ culture model was performed using the aortic ring assay. As described previously [31], thoracic aortas were excised from 4-week old mice, flushed with media to remove the clotted blood inside, and the periadventitial fibroadipose tissue trimmed away. Excised aortas were inverted to expose the vascular endothelium, cut into small cross-sectional pieces approximately 1 mm long, and placed into a well of a 8-chamber slide (Lab-Tek™, NUNC, Rochester, NY) that had been pre-coated with a 0.1 mg/ml collagen solution (Stemcell Technologies, Vancouver, BC, Canada). The aortic rings were incubated in either experimental or control co-culture supernatants for 48 hrs at 37°C and 5% CO_2_. Endothelial outgrowths of the cultured rings were photographed and the number of cell nuclei counted. At least 6 replicates were used in each group.

### Microglial Chemotaxis Assay

Microglial chemotaxis was evaluated using 48-well Boyden chemotaxis chambers (Neuroprobe, Gaithersburg, MD) [32]. A 5 µm polycarbonate filter (Neuroprobe, Gaithersburg, MD) separated the top and bottom chamber compartments. Cultured retinal microglia cells growing in a culture flask were harvested with 0.25% trypsin-EDTA, neutralized with 10% serum in DMEM medium, washed 3 times with serum-free DMEM medium, and resuspended in DMEM plus 5% heat inactivated serum to a concentration of 1-2×10^5^ cells/ml. A volume of 50 µl of resuspended cells was added to the top chamber and a volume of 50 µl of experimental or control medium was added to bottom chamber. The chamber assembly was then incubated for 2 hrs at 37°C. On disassembly of the chamber, the membrane filter was wiped off carefully to remove adherent cells on the top of the filter, fixed in 4% paraformaldehyde for 30 min, and then washed 3 times with PBS. Cells migrating to the bottom of the filter were stained with DAPI (Vector Labs, Burlingame, CA), photographed with an epifluorescence microscope, and counted.

### Subretinal Injection of Retinal Microglia

Experimental animals were anesthetized by the intraperitoneal injection of ketamine (90 mg/kg) and xylazine (8 mg/kg). As described in Zhao et al.[33], the sclera was exposed on the temporal side of the globe and a small incision made with a sharp 33-gauge needle between the limbus and the equator to gain access to the subretinal space. Cultured microglia, previously been activated with LPS (1 µg/ml) for 6 hrs, and then washed 3 times in HBSS, were resuspended in DMEM medium at a density of 1×10^8^/ml. This suspension was then added to growth factor-reduced Matrigel (BD Biosciences, Bedford, MA) at a 1∶1 volume ratio and 1.5 µl of the resulting mixture was slowly injected through the scleral incision into the subretinal space using a blunt needle attached to a Hamilton micro-syringe. The injection was gradually introduced allowing the gel mixture to dissect smoothly into the subretinal space, forming a circular bleb posterior to the injection site. Control injections of a similar volume of Matrigel and DMEM medium (without microglia) were made into the subretinal space of the contralateral eye.

### Labeling of Choroidal Blood Vessels and Neovascularization

Experimental animals were euthanized with CO2 inhalation and perfused intracardiacally with 4–5 mls of PBS, followed by 4–5 mls of a 160 mM solution of 1, 1'-Dioctadecyi-3, 3, 3′, 3′-tetramethylindocarbocyanine perchlorate (DiI, Sigma-Aldrich, St. Louis, MO), and then finally with 20 mls of 4% paraformaldehyde (in 0.1 M phosphate buffer, pH 7.4), as previously described [34]. Eyes were harvested and sclerochoroidal whole-mounts of entire posterior segments prepared. The injection entry site and the area covered by the subretinal Matrigel-cell mixture were located on the whole-mounted tissue, photographed with an epifluorescence microscope and/or confocal microscopy (SP2; Leica, Exton, PA), and quantified using computer-assisted software (NIH Image J).

### Statistical Analysis

Statistical analyses were performed using GraphPad Prism Software (La Jolla, CA). Unpaired t-tests or analysis of variance (ANOVA) were used to determine whether differences existed between experimental mean values. A P value<0.05 was considered significant.

## Results

### Culture of Mouse Microglia Cells

**Figure 1:**
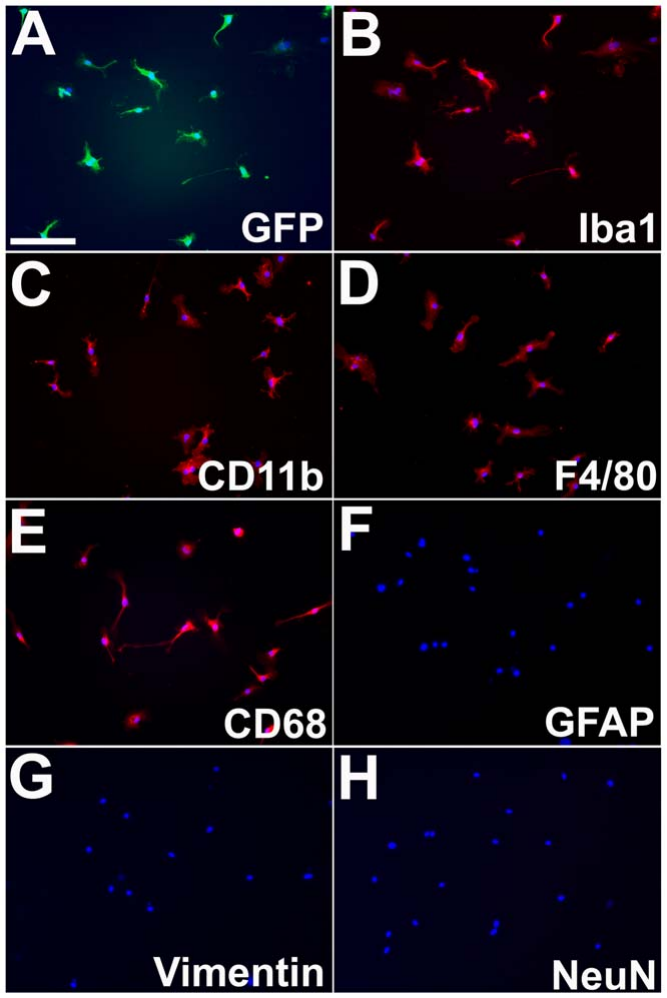
Immunoreactivity of cultured retinal microglia from CX3CR1^+/GFP^ transgenic mice. Retinal microglia isolated from CX3CR1^+/GFP^ transgenic mice exhibited GFP fluorescence (A) as driven by the microglial-specific CX3CR1 promoter. Cultured cells were immunoreactive for classic microglial markers Iba-1 (B), CD11b (C), F4/80 (D), and CD68(E). The cells were immunonegative for other macroglial markers such as GFAP (F), vimentin (G), and neuronal markers, such as NeuN (H). Scale bar = 100 µm.

Our procedure for isolating microglia in culture resulted in a population of cells that are highly enriched in retinal microglia. Almost all (>98%) of the DAPI-positive cells isolated from CX3CR1^+/GFP^ transgenic mice were also GFP-positive, indicating the expression of CX3CR1, a specific marker of microglia and dendritic cells in the retina [15] and the brain [35] (Fig. 1A). These GFP+ cells were also immunoreactive for markers positive in microglia including ionized calcium-binding adapter molecule-1 (Iba-1) (Fig. 1B), CD11b (Fig. 1C), F4/80 (Fig. 1D), and CD68 (Fig. 1E). Cultured cells were immunonegative for macroglial markers, such as glial fibrillary acidic protein (GFAP) (Fig. 1F) and vimentin (Fig. 1G), and for neural markers such as NeuN (Fig. 1H) and neurofilament-associated antigen, 3A10 (data not shown). These findings indicate the predominance of microglia cells in the culture with a relative absence of contaminating glial and neuronal cells.

### Co-culture with Retinal Microglia Induces Structural and Proliferative Changes in Primary RPE Cells

In order to investigate the effects of activated retinal microglia on RPE cells, we employed an *in vitro* system in which primary mouse RPE cells are grown to confluence in culture and then co-cultured with mouse retinal microglia cells (RMG) isolated from animals of the same genetic background. Retinal microglia were activated by exposure to LPS (1 µg/ml for 6 hrs), washed extensively with media, and then co-cultured with primary RPE cells on an adjoining tissue-culture insert for 24 hrs, without directly exposing target RPE cells to LPS. RPE cells co-cultured with unactivated RMG and RPE cells which were unexposed to RMG were used as controls. After co-culture, the RPE cells and the overlying supernatant are harvested and analyzed separately.

**Figure 2:**
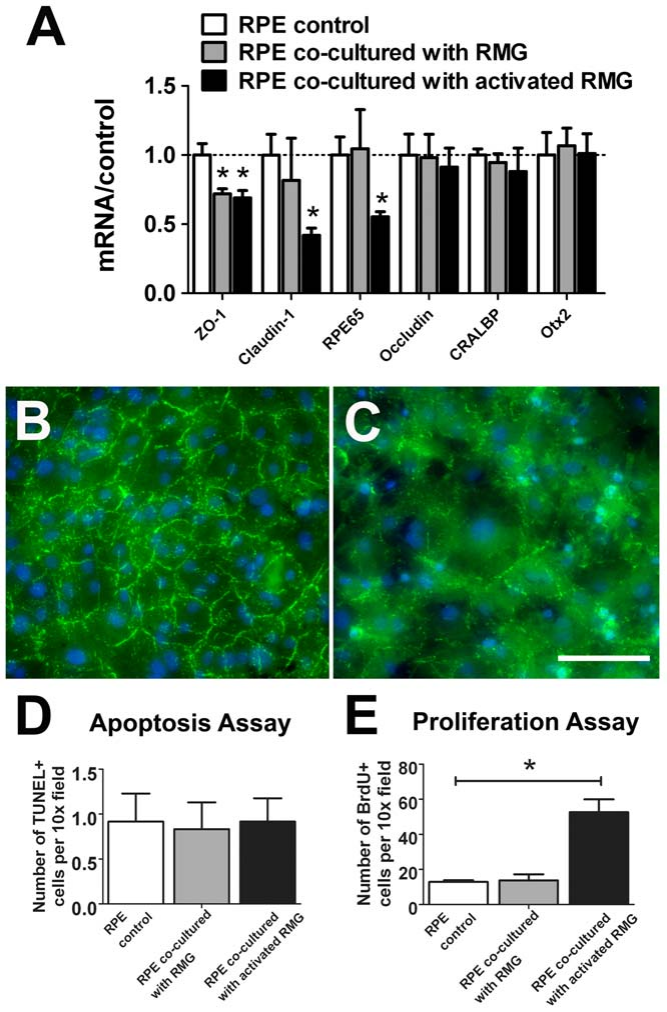
Primary RPE cells are altered in gene expression, structure and proliferation following co-culture with retinal microglia (RMG). (A) Quantitative RT-PCR comparing mRNA levels of visual cycle (RPE65, CRALBP) and junctional proteins (ZO-1, Claudin-1, Occludin) in RPE cells cultured alone (control, white bars), or co-cultured with either unactivated RMG (gray bars) or RMG pre-activated with LPS (black bars). Results were normalized relative to levels in unexposed RPE cells. (B–C) ZO-1 labeling (green) in confluent primary RPE cells co-cultured in the absence (B) and the presence of activated RMG (C). Co-culture with RMG induced the loss of discrete localization of ZO-1 in RPE tight junctions. Scale bar = 50 µm. (D) RPE cell apoptosis, as measured by TUNEL labeling, in the presence of conditioned media from unexposed RPE cells (white), or from RPE cells co-cultured with unactivated (gray), or activated RMG (black) (*p = <0.0001, n = 8 replicates). (E) RPE cell proliferation, as measured by BrdU staining, in the presence of unexposed RPE cells (white) or from RPE cells co-cultured with unactivated RMG (gray), or activated RMG (black). (p = 0.973, n = 12 replicates for each condition.) Significant differences (p<0.05) are indicated with *.

We first investigated the effect of RMG co-culture on RPE cells in terms of their expression of visual cycle proteins and structural junctional proteins. RPE cells co-cultured with LPS-activated microglia, compared to unexposed RPE cells, expressed significantly lower levels of junctional proteins ZO-1 and claudin-1, as well as lower amount of RPE-65 (Fig. 2A). RPE cultured with unactivated RMG expressed significantly reduced levels of ZO-1, but not claudin-1 and RPE65, when compared with unexposed controls. However, mRNA levels of occludin, CRALBP, and RPE-transcription factor, Otx2, were unchanged with microglia co-culture. These induced alterations in gene expression in RPE were accompanied by significant changes in the structure of the primary RPE cells. Immunostaining for ZO-1, a protein normally located in the tight junctions between RPE cells, revealed a marked mislocalization of ZO-1 from a junctional distribution seen in control cultures (Fig. 2B) to a more diffuse, cytoplasmic pattern following microglia co-culture (Fig. 2C), suggesting a disorganization of tight junctions. The regular hexagonal mosaic of RPE cells was also disrupted, with individual RPE cells showing pleomorphic morphologies and irregular distributions, indicating a loss of proper cell-cell relationships between neighboring RPE cells.

The effect of microglia co-culture (either with or without prior-LPS activation) however did not result in a higher level of apoptotic RPE cell death as demonstrated using a TUNEL assay (Fig. 2D). Despite prominent structural disorganization, very few RPE cells following microglia co-culture were TUNEL-positive. Conversely, RPE cells co-cultured with LPS-activated microglia demonstrated significantly higher rates of proliferation, with greater numbers of cells showing positivity for BrdU labeling, relative to either unactivated microglia co-culture or unexposed RPE cells, (Fig. 2E). In summary, exposure to activated microglia induced RPE cells to change their expression of certain tight junction and visual cycle proteins, to lose the integrity of tight junctions between neighboring cells, and to increase in their proliferation rate, that in combination is likely to have contributed to a more disorganized and irregular RPE layer.

### RPE Expression of Chemotactic Cytokines and Cell Adhesion Molecules Is Increased Following Co-Culture with Activated Retinal Microglia

**Figure 3:**
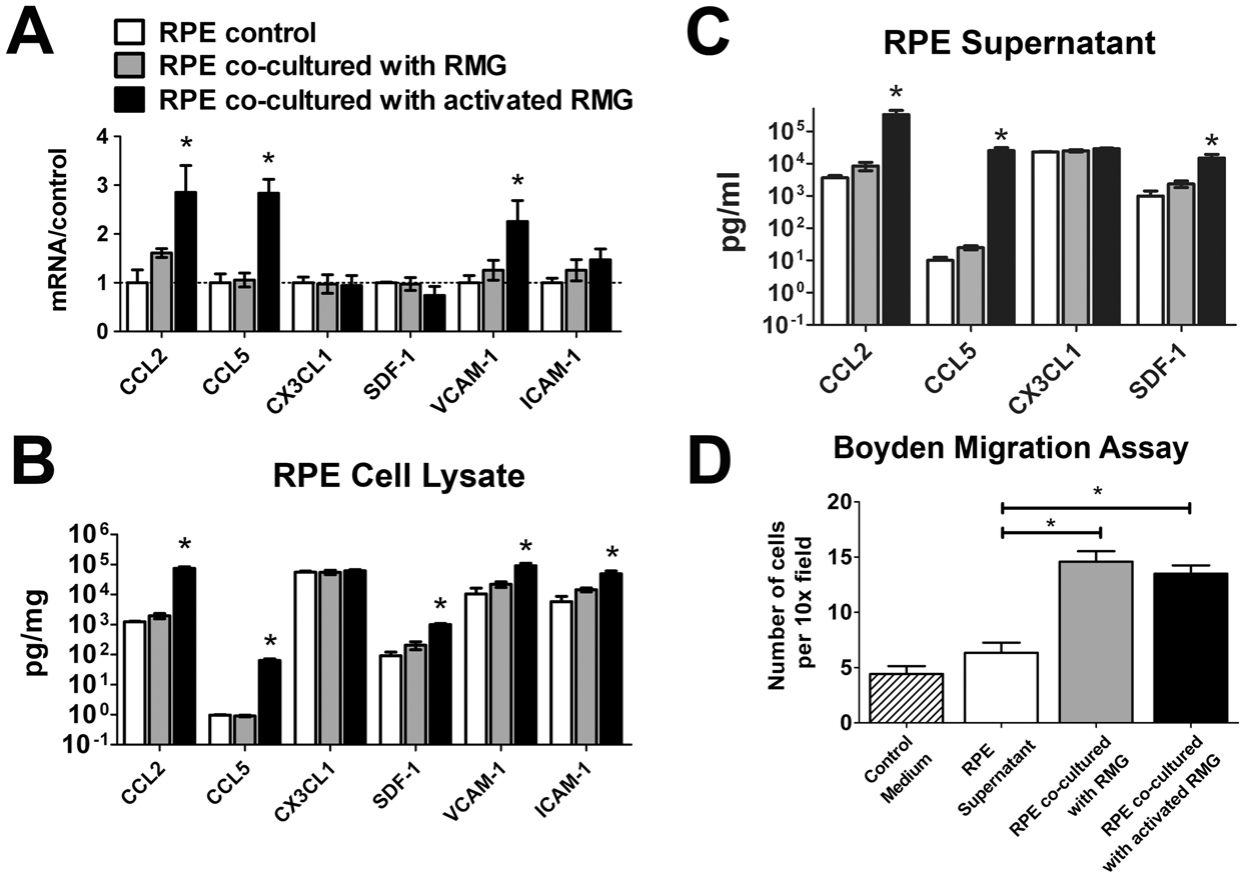
Primary RPE cells increase expression and secretion of chemotactic cytokines and cell adhesion molecules following co-culture with retinal microglia (RMG). (A) Quantitative RT-PCR comparing mRNA levels of chemotactic cytokines (CCL2, CCL5, CX3CR1, SDF-1), and adhesion molecules (VCAM-1, ICAM-1) in RPE cells. Levels from RPE cells co-cultured with unactivated RMG (gray bars) or activated RMG (black bars) were measured and normalized relative to levels from unexposed RPE cells (white bars). Levels of chemokines and adhesion molecules in the cell lysates (B) and supernatants (C) of RPE cultures were measured with ELISA and compared. Chemotactic cytokines, CCL2, CCL5, and SDF-1, capable of attracting microglia cells, were found at increased levels in supernatant of RPE cells co-cultured with activated RMG. These chemokines were also slightly but non-significantly, elevated in the presence of unactivated RMG. RPE cell lysates also contained increased levels of the adhesion molecules VCAM-1 and ICAM-1 that are able to bind and retain microglia in the subretinal space. (D) Chemotaxis of retinal microglia, as assayed using a Boyden chemotaxis chamber, was significantly increased when incubated with supernatant from RPE cultures co-cultured with either unactivated (gray bar) or activated RMG (black bar), when compared to that from unexposed RPE cells or to control medium (DMEM). (*p = <0.001, n = 12 replicates). Significant differences (p<0.05) are indicated with *.

In aged animals, microglia accumulates in the subretinal space as a function of increasing age [16]. It is possible that microglia in the subretinal space may induce changes that may further attract additional microglia cells to the area. To address the question of whether RMG-induced changes in RPE cells may result in the further microglial recruitment, we assayed the expression of chemotactic cytokines, CCL2 (MCP1), CCL5 (RANTES), CX3CL1 (fractalkine), and SDF-1 (CCL12), in RPE cells that were either co-cultured in the presence of activated RMG, unactivated RMG, or cultured alone. We also assayed the RPE expression levels of membrane-associated adhesion molecules, VCAM-1 and ICAM-1, which can bind receptors on the cell surfaces of microglia and retain them in the subretinal space. We found that the transcription levels of CCL2, CCL5, and VCAM-1 mRNA were significantly increased after co-culture with activated retinal microglia compared to unexposed controls (Fig. 3A). The level of ICAM-1 mRNA was slightly but not significantly increased. Protein levels of CCL2, CCL5, as well as SDF-1, were also found on ELISA analysis to be elevated in both RPE cell lysates and supernatants when co-cultured with activated RMG (Fig. 3B, C). VCAM-1 and ICAM-1 levels were also both increased in RPE cell lysates of these cultures. However, CX3CL1 expression in RPE cells was not altered by activated RMG co-culture (Fig. 3A–C). In general, the levels of chemokine expression in RPE cells co-cultured with unactivated RMG were either similar to that in unexposed RPE controls or intermediate between that found in unexposed controls and RPE cells co-cultured with activated RMG (gray bars, Fig. 3A–C). Taken together, co-culture with activated retinal microglia induced RPE cells to up-regulate the expression and secretion of chemoattractants, CCL2, CCL5, and SDF-1, which are capable of attracting retinal microglia into the subretinal space, and to also increase the expression of cell-surface adhesion molecules which may retain microglia in that location.

Supernatants from RPE co-cultures were also assessed for their ability to induce microglia chemotaxis using a Boyden chamber assay. While supernatants from unexposed RPE cells did not significantly increase the number of migrating microglia compared to control medium (DMEM) (Fig. 3D), supernatants from RPE cells previously co-cultured with either unactivated or activated retinal microglia were able to significantly augment microglia migration. This indicated that retinal microglia, independent of their previous activation by LPS, can induce RPE cells to produce factors effective in increasing the chemotaxis of retinal microglia as assessed in this *in vitro* assay.

### RPE eExpression and Secretion of Pro-Inflammatory Cytokines Is Increased Following Co-Culture with Activated Retinal Microglia

**Figure 4:**
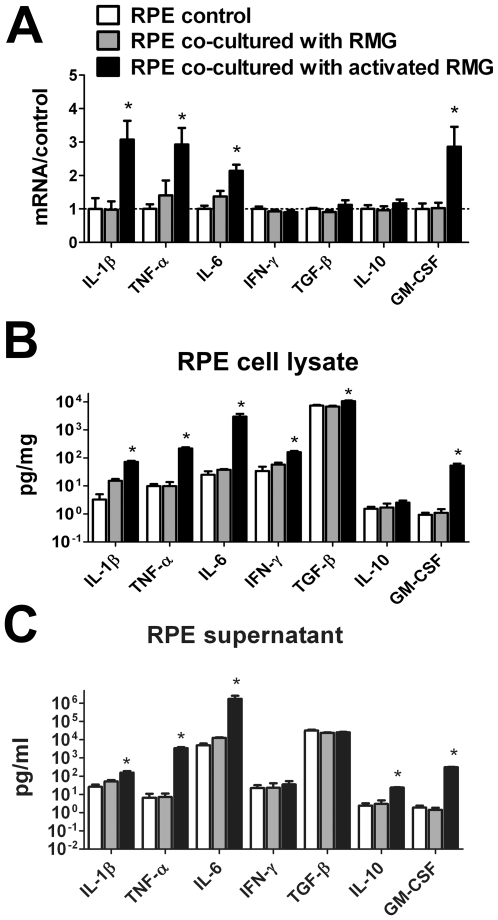
Primary RPE cells increase expression and secretion of pro-inflammatory cytokines following co-culture with retinal microglia (RMG). (A) Quantitative RT-PCR comparing mRNA levels of pro-inflammatory factors (IL-1β, TNF-α, IL-6, IFN-γ, GM-CSF) and anti-inflammatory factors (IL-10, TGF-β) in RPE cells co-cultured with unactivated (gray bars) and activated (black bars) RMG relative to unexposed RPE cells (control, white bars). Results were normalized relative to levels in unexposed RPE cells. Levels in the cell lysates (B) and supernatants (C) of RPE cultures were also measured with ELISA and compared. RPE expression of pro-inflammatory cytokines IL-1β, TNF-α, IL-6, and GM-CSF were substantially elevated following culture in both cell lysate and supernatants, reflecting a general increase in pro-inflammatory mediators. Significant differences (p<0.05) are indicated with *.

Previous studies have demonstrated that RPE cells are able to express and secrete various pro-inflammatory and anti-inflammatory factors, often under the influence of various cytokines [36]. We investigated here the effect of retinal microglia co-culture on the expression of inflammatory factors and cytokines in RPE cells. We observed that relative to controls, RPE co-cultured with activated RMG expressed higher mRNA levels of the pro-inflammatory factors, IL-1β, TNF-α, IL-6, and GM-CSF (Fig. 4A). ELISA analyses also revealed that the levels of these factors in the RPE cell lysates (Fig. 4B) and RPE supernatants (Fig. 4C) were markedly increased. IFN-γ levels were also increased in the cell lysate but not in the supernatant. Conversely, the anti-inflammatory cytokine, IL-10, which was present at low levels in the RPE cells, was not markedly changed. Expression levels of TGF-β, which are constitutively high in unexposed RPE cells, did not markedly alter after co-culture with activated RMG. In general, the levels of cytokine expression in RPE cells co-cultured with unactivated RMG were either similar to that in unexposed RPE controls or intermediate between that found in unexposed controls and RPE cells co-cultured with activated RMG (gray bars, Fig. 4A–C). Overall, the effect of co-culture with activated microglia increased the RPE expression and secretion of multiple pro-inflammatory cytokines, suggesting that the subretinal accumulation of microglia in aged animals may promote a more pro-inflammatory environment in the subretinal space.

### RPE Expression and Secretion of Pro-Angiogenic Mediators Is Increased Following Co-Culture with Activated Retinal Microglia

**Figure 5:**
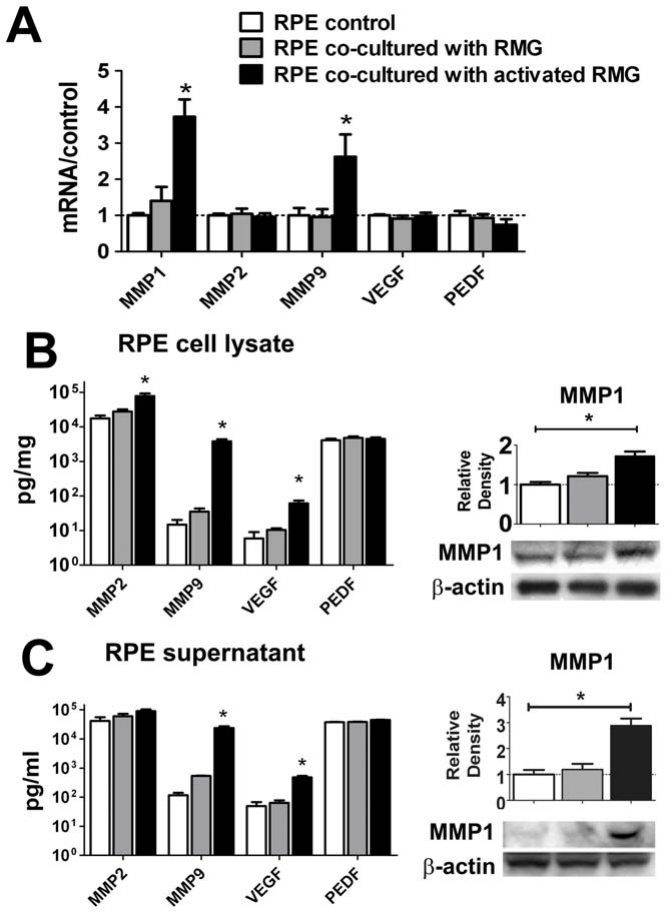
Primary RPE cells increase expression and secretion of pro-angiogenic factors following co-culture with retinal microglia (RMG). (A) Quantitative RT-PCR comparing mRNA levels of metalloproteinases (MMP1, MMP2, MMP9), and growth factors, VEGF and PEDF, in RPE cells co-cultured with unactivated (gray bars) and activated RMG (black bars) relative to control (unexposed RPE cells, white bars). (B) Protein analyses of RPE cell lysates showing that levels of MMP2, MMP9, and VEGF, as analyzed by ELISA, were increased following RMG co-culture (left). MMP1 levels, as analyzed by Western blotting, were also significantly elevated (right). (C) Protein analyses of RPE supernatants showing similar significant increases by ELISA analyses (MMP9, VEGF, left) and by Western blotting (MMP1, right). Levels of PEDF, an anti-angiogenic factor, were unchanged by co-culture in both RPE cell lysates and supernatants. Significant differences (p<0.05) are indicated with *.

RPE expression levels of angiogenic mediators were also assessed at the mRNA and protein levels in the presence and absence of retinal microglia co-culture. In particular, the expression of metalloproteinases was assessed. These included MMP1 (interstitial collagenase), MMP2 (gelatinase A), and MMP9 (gelatinase B), whose ability to degrade extracellular matrix components may promote endothelial cell migration and subretinal neovascularization. Also, expression levels of the pro-angiogenic factor, vascular endothelial growth factor (VEGF), and the anti-angiogenic factor, pigment epithelial growth factor (PEDF), were evaluated. Figure 5 shows that mRNA levels were increased for MMP1 and MMP9 (Fig. 5A), while protein levels of MMP1, MMP2, and MMP9, as well as VEGF, as assessed by ELISA and Western blot analysis, were increased in RPE cell lysates (Fig. 5B) and supernatants (Fig. 5C) following co-culture with activated microglia. PEDF expression and secretion levels were however relatively unchanged. In general, the levels of angiogenic factors in RPE cells co-cultured with unactivated microglia were either similar to that in unexposed RPE controls or intermediate between that found in unexposed controls and RPE cells co-cultured with activated microglia (gray bars, Fig. 5A–C). These results indicate that activated microglia in the subretinal space may induce expression and secretion changes in RPE cells that can foster a more pro-angiogenic local environment at the retinochoroidal junction.

**Figure 6:**
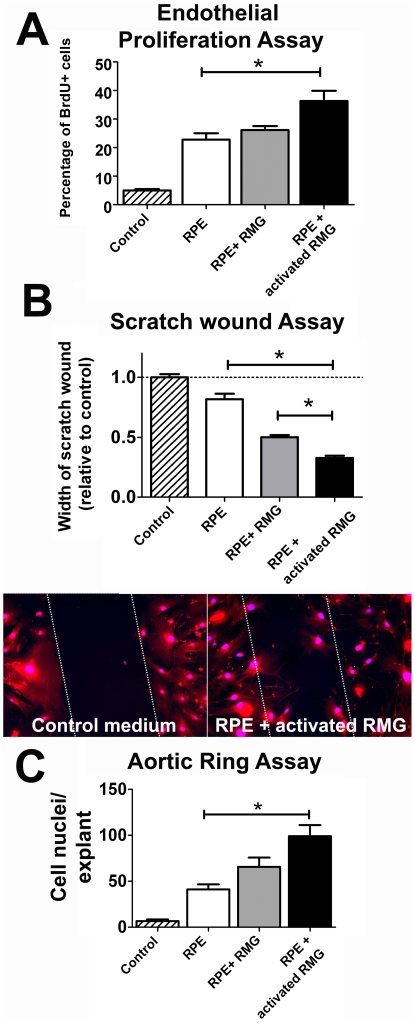
Conditioned supernatants from RPE cells co-cultured with retinal microglia (RMG) increase angiogenesis in *in vitro* models. (A) Primary endothelial cells show higher rates of proliferation when exposed to supernatants of RPE cells co-cultured with activated RMG (black bar) compared to supernatants from unexposed RPE cells (white bar) (*p = 0.007, unpaired t-test, n = 7 replicates). Proliferation of endothelial cells exposed to supernatants of RPE cells co-cultured with unactivated RMG (gray bar) was slightly but not significantly elevated. (B) Primary endothelial cell migration, as evaluated by a scratch wound assay, was increased in the presence of culture supernatants from RPE cells co-cultured with either unactivated (gray bar) or activated RMG (black bar), relative to that from unexposed RPE (white bar). The assay was scored according to the ability of endothelial cells to migrate into a scratch wound of uniform width marked on a confluent layer of endothelial cells (lower panels, cells stained with CD31 (red) and DAPI (blue), n = 9 replicates) (C) Sprouting angiogenesis, as evaluated by an aortic ring assay, was increased in the presence of culture supernatant from RPE cells co-cultured with activated RMG (black bar), compared to that from unexposed RPE (white bar). Measurements were slightly but non-signficantly increased with supernatants from RPE cells co-cultured with unactivated RMG (gray bar). Endothelial cell nuclei from vessels sprouting from sections of vascular aortic rings were scored (n> = 6 replicates).

The ability of these microglia-induced alterations to increase angiogenesis was also evaluated in a number of *in vitro* assays. Supernatants from RPE cells co-cultured with activated microglia, with unactivated microglia, and from unexposed RPE cells, were placed together with primary endothelial cells or endothelial organ cultures and tested for their ability to affect angiogenesis. Supernatants from activated microglia-RPE co-cultures significantly increased primary endothelial cell proliferation relative to those from unexposed RPE cells as measured using a BrdU assay (Fig. 6A). Also, these supernatants promoted a relatively higher rate of primary endothelial cell migration as evaluated using a scratch wound assay (Fig. 6B). Sprouting angiogenesis as evaluated in an organ culture (aortic ring) assay was also significantly increased in the presence of supernatants from activated microglia co-cultures (Fig. 6C). Generally, the effects of supernatants from unactivated microglia-RPE co-cultures were intermediate between those that induced by supernatants from unexposed RPE cultures and activated microglia-RPE co-cultures. These findings underline the ability of activated RMG to induce RPE changes that promote angiogenesis.

### Subretinal Transplantation of Retinal Microglia Promotes Choroidal Neovascularization, RPE Disorganization, and Microglia Recruitment

**Figure 7:**
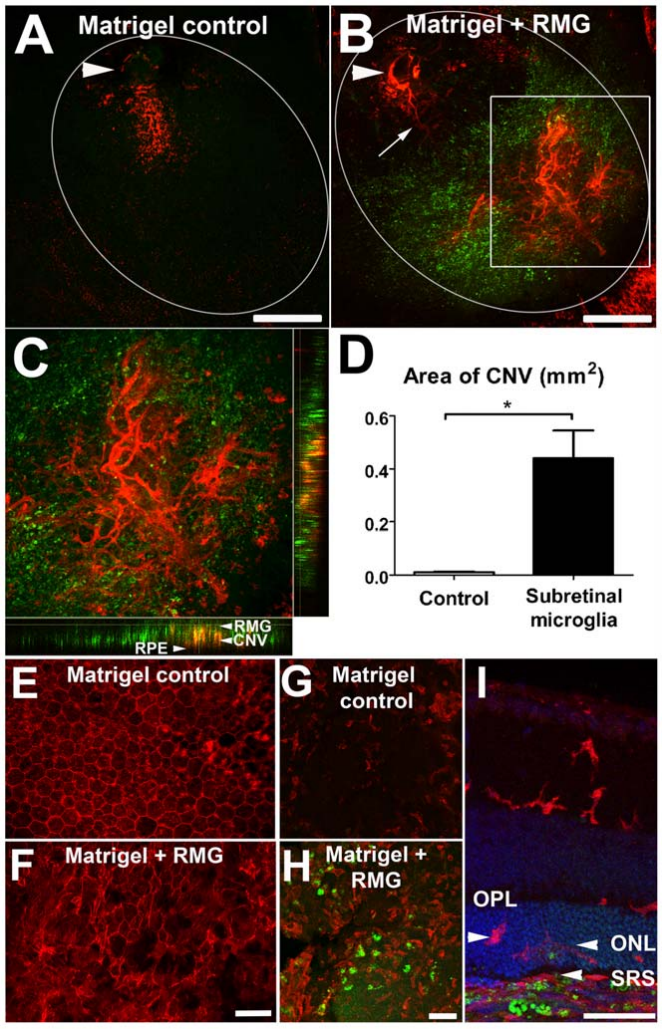
*In vivo* subretinal transplantation of retinal microglia promotes choroidal neovascularization, RPE disorganization, and microglia recruitment. The tissue effects of juxtaposing microglia with RPE cells were assessed in an *in vivo* mouse model by the injection of cultured microglia with factor-reduced Matrigel into the subretinal space of adult, wild-type mice. Injected microglia, derived from CX3CR1^+/GFP^ mice, were marked by GFP expression. Contralateral eyes in experimental animals served as controls (injected with factor-reduced Matrigel only without microglia). Four days after transplantation, mice were euthanized and perfused with DiI to mark vascular structures. (A) Representative RPE flatmount from a control eye. Arrowhead marks the injection entry site, and ellipse outlines the area of the subretinal bleb formed. Little or no associated CNV (marked in red by DiI perfusion) formation were observed in control eyes. (B) Representative RPE flatmount from an eye injected with a subretinal injection of Matrigel containing microglia (green). DiI perfusion reveals presence of a feeding vessel (arrow) growing from the injection entry site (arrowhead) to form a branching CNV complex that is centered at the site of microglia cell accumulation. Scale bar (A, B) = 300 µm (C) Magnification of CNV complex (inset in B) showing location of the CNV complex between the RPE and the overlying microglia in the subretinal space (side panels). (D) Quantification of the area of CNV membranes formed in control eyes versus eyes with subretinal microglia showed a significant increased in CNV formation (n = 10 animals, p<0.003, paired t-test). (E–F) Phalloidin staining of F-actin in RPE flat-mounts in the area of subretinal injection. The RPE layer in Matrigel controls demonstrated a predominantly regular array of uniformly hexagonal cells with normal cell-cell relations (E), while that in the area of subretinal microglia transplantation showed pleomorphic and irregularly-spaced cells in which the location of F-actin appeared disorganized (F). (G–H) Iba-1 staining of microglia in the outer retina (retinal flat-mounts, mounted photoreceptor-side up) in control (G) and microglia-transplanted eyes (H). The recruitment of endogenous microglia (labeled in red only) to the outer retina was increased in the region of transplanted GFP+ microglia (green). (I) Migration of endogenous microglia (red) was observed from the inner retina to the outer nuclear layer (ONL) and subretinal space (SRS) (arrowheads) in the area of subretinal microglia transplantation (green). Scale bars (E–I) = 50 µm.

In order to evaluate how the *in vivo* presence of subretinal microglia may contribute to a pro-inflammatory, pro-angiogenic environment, activated retinal microglia were transplanted into the subretinal space above the RPE layer. GFP-labeled retinal microglia, isolated from CX3CR1^+/GFP^ mice, were activated with LPS, washed extensively, placed in growth-factor reduced Matrigel, and then injected into the subretinal space of adult wild-type mice. Injections of growth-factor reduced Matrigel mixed with DMEM medium (without microglia) were performed in the contralateral eyes to serve as controls. Animals were euthanized 4 days following subretinal injection and perfused with DiI to label choroidal vascular structures. Analysis of RPE flatmounts revealed that eyes with subretinally-transplanted microglia develop large and prominent choroidal neovascularization (CNV) membranes in the subretinal space, whereas CNV membranes were either absent or much more rudimentary in control injections (Figure 7A–D). The CNV membranes that formed in treated eyes were observed to extend horizontal in a branching manner in the subretinal space within the zone of subretinal microglia transplantation (Fig. 7C).

RPE cells underlying the area of subretinal microglial transplantation were also evaluated in sclerochoroidal whole-mount preparations. Phalloidin staining for F-actin in RPE cells revealed that while regular structure and distribution of RPE cells were mostly preserved in control injections (Fig. 7E), the structure of RPE cells that were in contact with injected subretinal microglia were markedly more pleomorphic and irregularly distributed (Fig. 7F), similar to that seen in co-cultured primary RPE cells. Quantitative comparisions of the fractional area of the RPE layer with abnormal RPE cell distributions also showed significant differences between control and microglia-injected eyes (data not shown). In addition, endogenous (i.e., non-transplanted) retinal microglia in recipient animals were immunolabeled with Iba-1 in order to follow changes in their distribution after transplantation. We observed that these GFP-, Iba-1+ retinal microglia were recruited to the outer retina at the injection site in greater numbers than that seen in control eyes (Fig. 7G and H). These cells were likely to have migrated from the inner retina through the outer nuclear layer to gain access to the subretinal space where they subsequently accumulate (Fig. 7I).

## Discussion

In the present study, we found that as a consequence of coming into close proximity with activated retinal microglia, primary RPE cells developed multiple structural and functional alterations. These involved decreased expression levels of visual cycle protein, RPE65, and proteins in the tight junctions (ZO-1 and claudin-1), a disruption of ZO-1 localization in cell junctions, and a loss of regular RPE cell shape and distribution. Interestingly, RPE cells co-cultured with retinal microglia did not undergo higher rates of apoptosis but instead exhibited a higher proliferative capacity. These changes indicate that RPE cells, under the influence of activated microglia, may lose integrity in their cellular morphology and intercellular contacts, proliferate in a less regulated manner, and thus lose its original configuration as a uniformly-spaced monolayer and form irregular cellular aggregates as seen in our *in vitro* and *in vivo* experiments. Indeed, in eyes with early and intermediate AMD, prior to the onset of CNV, analogous changes, the form of RPE hypertrophy and clumping in the subretinal space and outer retina seen may be seen on histopathological and clinical examination [7], [8]. Photoreceptor loss [8] and synaptic pathology [37] have also been observed in AMD eyes in areas of drusen and pigmentary alteration, which may potentially be related to decreases in expression of RPE65 in RPE cells, inducing dysfunctional changes in visual pigment cycling and photoreceptor physiology [38]. Taken together, the changes induced by retinal microglia on RPE cells in our *in vitro* and *in vivo* models bear resemblances to aspects of RPE alterations in AMD, suggesting that the *in vivo* accumulation of retinal microglia in the subretinal space seen in AMD[19] may indeed drive relevant pathogenic mechanisms. In the late atrophic form of AMD, RPE cells undergo eventual atrophy in a contiguous manner in the form of geographic atrophy [39]; while we did not observe an increase in RPE apoptosis over the time scale of our *in vitro* co-culture systems, the possibility that prolonged co-culture with retinal microglia may result in pro-apoptotic effects cannot be ruled out.

RPE cells play an important immunomodulatory role in the outer retina, in part by producing and secreting multiple cytokines [36] that contribute to the environment of immune privilege in the subretinal space [13]. RPE cells, in expressing receptors for various cytokines [40], also respond prominently to cytokine signaling by altering levels of cytokine production and secretion, synthesizing nitric oxide [41], increasing adhesion molecule expression[42], and regulating RPE tight-junction integrity [43]. As LPS-activated retinal microglia secrete prominent levels of chemokines and inflammatory mediators [25], [44], the effects that microglia co-culture exert on RPE cells are likely mediated by chemokine signaling from retinal microglia. Among these changes is the up-regulation of cytokines that are strongly chemotactic for microglia, macrophages, and monocytes, such as CCL2, CCL5, and SDF-1. These factors likely contribute to the ability of co-culture RPE supernatants to promote the *in vitro* migration of microglia cells, and to recruit endogenous microglia *in vivo* to the subretinal space following microglia transplantation. These observations raises the possibility of a positive feedback mechanism by which early subretinal microglia accumulation, through induced changes in RPE gene expression and cytokine secretion, fosters a more pro-inflammatory and chemoattractive environment in the subretinal space. The further recruitment of microglia to the subretinal space, and their subsequent activation in that locus, perpetuate a progressive accumulation of microglia that incrementally abrogates the zone of immune privilege in the subretinal space and advances AMD-relevant pathogenic mechanisms at the retinochoroidal interface.

Our results showed that even though changes in RPE gene expression were larger in co-cultures with activated microglia than with unactivated microglia, the inductive effects of microglia without overt activation may still be functionally appreciable, such as in assays for microglial chemotaxis (Fig. 3D) and in vitro angiogenesis (Fig. 6B). However, it is likely that in the context of AMD, the displacement of retinal microglia to the subretinal space may also bring microglia into contact with influences that may serve to activate them. Retinal microglia observed in human AMD specimens have amoeboid morphologies, suggesting their activated status [19]. The initiating steps in this proposed model of cumulative subretinal microglia accretion have not been defined but may potentially be related to complement activation [45], drusen accumulation [46], photoreceptor injury [47], or oxidative stress in the outer retina [48]. Further studies on the regulatory mechanisms underlying microglia distribution and migration [24], [49] in the retina, as well as their activation in the subretinal space, may be useful in this respect.

Our results also indicated that retinal microglia co-culture increased in RPE cells the expression and secretion of VEGF and pro-angiogenic metalloproteinases, MMP1, MMP2, and MMP9. Co-culture RPE supernatants were also effective in increasing endothelial cell proliferation and migration as evaluated in 3 separate *in vitro* assays. These findings, together with an increase in pro-inflammatory and chemoattractive factors, suggest that microglial accumulation in the subretinal space may create a pro-angiogenic environment that increases the likelihood of the development of CNV. Metalloproteinases, thought to be capable of comprising the natural barrier function of Bruch's membrane to CNV growth [50], have been previously related to the formation of CNV in AMD and in animal models [51]–[54]. The growth of CNV may be further encouraged by the increased secretion of VEGF by RPE cells following co-culture that can directly stimulate endothelial cell proliferation and migration [55]. In support of the relationship betwen RPE alterations and CNV formation, epidemiological studies have also linked the clinical presence of RPE changes (scored as pigment abnormalities in the fundus) occurring in patients with early and intermediate AMD with an elevated 5-year risk for the development of CNV [10].

Our experiments with the *in vivo* transplantation of retinal microglia demonstrated that the presence of subretinal microglia exerts a strong pro-angiogenic influence in the formation and growth of CNV. The creation of a pro-angiogenic environment in the subretinal space, while mediated significantly by RPE alterations, may also be contributed towards by the subretinal microglia themselves. In our *in vitro* co-culture experiments, while mRNA and protein analysis in RPE cell lysates relate directly to RPE gene expression, protein analyses involving RPE co-culture supernatants may however also contain contributions secreted from retinal microglia cells. However, in our angiogenesis and microglia-migration functional assays, we found that the changes induced by RPE co-culture supernatants were significant when compared not only to unexposed RPE supernatants, but also when compared to supernatants of activated retinal microglia not exposed to RPE cells (data not shown). As such, despite possible contributions by retinal microglia themselves, changes in RPE gene expression and protein secretion are likely play a prominent role in inducing the structural and functional changes of pathological significance in the subretinal space.

While our *in vivo* model system permits direct cell-cell contact between retinal microglia and RPE cells, the *in vitro* system used here brings the two cell types in close proximity but however precludes direct cellular surface contact. The similarities in the nature of changes induced in both *in vitro* and *in vivo* systems (changes in RPE structure, increased chemotaxis of microglia, pro-angiogenic effects) suggest that many of these do not require direct microglial-RPE cell contact, although additional studies may be required to investigate the effects that result only from direct cellular contact. In addition to retinal microglia-to-RPE communication, other forms of intercellular interactions may also play a role in AMD pathogenesis. In producing multiple cytokines, RPE cells are likely to reciprocally affect physiology of subretinal microglia [56], [57]. The close anatomical proximity of drusen, which contain multiple immunologically active molecules [58], [59], may also contribute to other pathologically relevant effects. Factors secreted or released from photoreceptors [25] and Muller cells [60] in the vicinity may also contribute. Future studies on the details of the interactions between these cellular players converging in the subretinal space, and how these are modulated by drusen components, can help elucidate these cellular relationships further.

We also propose that the subretinal transplantation of cultured retinal microglia employed here constitutes a useful animal model for future study of intercellular and molecular interactions in this locus. To the extent that retinal microglia-RPE interactions may represent a potential therapeutic locus for intervention, this transplantation model may be useful in the evaluation of potential inhibitory agents [61]. Agents that successfully amelioriate the structural changes in RPE cells and/or the growth of CNV membranes into the subretinal space in this model may be promising candidates for AMD therapy and prevention.

In summary, our results indicate that retinal microglia, when placed in proximity to RPE cells, induce significant changes in 1) the structure and distribution of RPE, and 2) expression and secretion of multiple molecules from RPE cells that have significant effects on the activation, recruitment, and retention of immune cells and on angiogenesis. The *in vivo* presence of subretinal microglia, simulated here by the subretinal transplantation of cultured microglia, corroborate our *in vitro* findings, promoting choroidal neovascularization, RPE disorganization, and further microglia recruitment into the subretinal space. These histological features, reminiscent of those found in both the early and later exudative stages of AMD, suggest that the interaction between microglia and RPE cells in the subretinal space may have important relevance to the pathogenesis and progression of AMD.
